# Lunularin Producers versus Non-producers: Novel Human Metabotypes Associated with the Metabolism of Resveratrol by the Gut Microbiota

**DOI:** 10.1021/acs.jafc.2c04518

**Published:** 2022-08-18

**Authors:** Carlos E. Iglesias-Aguirre, Fernando Vallejo, David Beltrán, Elena Aguilar-Aguilar, Julio Puigcerver, Mateo Alajarín, José Berná, María V. Selma, Juan Carlos Espín

**Affiliations:** † Laboratory of Food & Health, Research Group on Quality, Safety, and Bioactivity of Plant Foods, CEBAS-CSIC, Campus de Espinardo, Murcia 30100, Spain; ‡ Nutrition and Clinical Trials Unit, GENYAL Platform, IMDEA-Food Institute, CEI UAM + CSIC, Madrid 28049, Spain; § Department of Organic Chemistry, Faculty of Chemistry, University of Murcia, Murcia 30100, Spain

**Keywords:** resveratrol, metabotype, gut microbiota, lunularin, interindividual variability

## Abstract

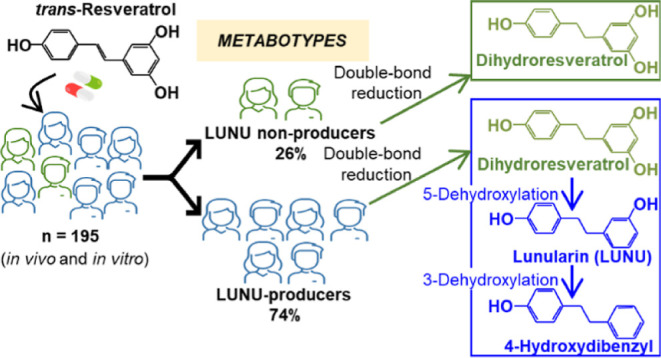

We describe here for the first time the consistent observation of two metabotypes associated with resveratrol metabolism by the human gut microbiota, that is, lunularin (LUNU)-producers and LUNU non-producers. In healthy volunteers (*n* = 195), resveratrol was reduced to dihydroresveratrol, which only in the LUNU-producer metabotype was sequentially dehydroxylated at the 5-position to yield LUNU and the 3-position to produce 4-hydroxydibenzyl. These metabolites (also 3,4′-dihydroxy-*trans*-stilbene in some LUNU-producers) were detected in the urine and (or) feces of 74% of volunteers after consuming resveratrol, while 26% lacked these dehydroxylase activities. The LUNU non-producer metabotype was more prevalent in females (*P* < 0.05) but independent of individuals’ BMI and age. A 4-styrylphenol reductase in both metabotypes converted stilbenes to their corresponding dibenzyls, while no 4-dehydroxylation in stilbenes or dibenzyls was observed. 4-Hydroxy-*trans*-stilbene, pinosylvin, dihydropinosylvin, 3-hydroxydibenzyl, and 3-hydroxy-*trans*-stilbene were not detected in vivo or in vitro. Further research on LUNU metabotypes, their associated gut microbiota, and their impact on health is worthwhile.

## Introduction

There is notable human interindividual variability in response to (poly)phenol consumption,^1,2^ and the two-way interaction between (poly)phenols and the gut microbiota is the primary driver of this interindividual variation.^3−8^ The metabolism of (poly)phenols by the gut microbiota gives rise to the so-called “high producers” and “low producers” of some metabolites whose production gradient is affected by external factors.^7^ This is the case for most (poly)phenols, including flavanones, anthocyanins, proanthocyanidins, lignans, and others.^2,7,9−12^ However, in other (poly)phenols, such as ellagic acid (EA) and isoflavones, the metabolism is characterized by particular gut microbial ecologies that yield specific metabolic phenotypes, that is, metabotypes. The term metabotype is an extensive concept involving individuals’ differential metabolic responses to nutritional or pharmacological interventions.^2,7^ Specifically, in the context of (poly)phenols’ metabolism and health, metabotype refers to a differential metabolic phenotype defined by specific metabolites derived from the gut microbiota, characteristic of the precursor (poly)phenol metabolism. A metabotype is also characterized by the associated microbial ecology in terms of composition and activity, which could notably impact human health. Therefore, this feature is less influenced by external factors and refers to a qualitative-genuine criterion (i.e., production vs non-production but not just high vs low metabolite excretion) concerning the specific microbial ecology that harbors each individual.^2,4,7^ In this context, the metabolism of EA and the isoflavone daidzein fulfills the definition of metabotype.^2,4,10,13,14^ In the case of EA, three urolithin-related metabotypes have been defined depending on the final urolithins produced, that is, metabotype A [3,8-dihydroxy-urolithin (urolithin A) producers], metabotype B [production of urolithins A and B (3-hydroxy-urolithin)], and (or) isourolithin A (3,9-dihydroxy-urolithin) and metabotype 0 (urolithin non-producers).^13,15^ In the case of isoflavones, the equol- and *O*-desmethylangolesin (ODMA)-producer metabotypes have been identified so far.^14,16^ In addition, a gradient of metabolite production may also occur within a specific metabotype, that is, high and low urolithin or equol producers.^7^ Overall, the potential biological activity derived from (poly)phenol intake is conditioned by the gut microbiome ecology of each individual.^5−7^ Therefore, the stratification, that is, grouping of individuals according to their gut microbiota (poly)phenol metabotypes, has been proposed to understand individuals’ responses to dietary (poly)phenols, that is, to explain their different metabolisms and health effects, which could be crucial in the context of personalized nutrition.^2,4,14,17,18^

Resveratrol (RSV) is the most relevant dietary stilbene due to its well-known bioactivity, including antioxidant, anti-inflammatory, immunomodulatory, anti-diabetic, cancer chemopreventive, neuroprotective, and cardiovascular protective effects.^19^ Relevant amounts of RSV can be consumed only through nutraceutical preparations since its presence in the human diet is limited to a few foodstuffs that contain low RSV levels, including grapes, red wine, peanuts, pistachios, and some types of berries (strawberries, blueberries, and others).^19^

The metabolism of RSV by the gut microbiota was previously reported to produce dihydroresveratrol (DHRSV), 3,4′-dihydroxydibenzyl (also known as lunularin, LUNU), and 3,4′-dihydroxy-*trans*-stilbene (DHST), showing high interindividual variability in the type of metabolites produced.^9^ However, this variability has been reported in a few volunteers or in vitro fecal samples.^9,20^

Recently, we have reported the presence of 4-hydroxydibenzyl (4HDB), also known as 4-(2-phenylethyl)phenol according to the IUPAC, as a novel metabolite from the human gut microbiota in the urine of healthy volunteers (*n* = 59) after consuming RSV.^21^ In that study, we observed that LUNU, but not DHST, was further dehydroxylated at the 3-position to yield 4HDB. We also observed substantial variability in the metabolism of RSV by the gut microbiota. Notably, 4HDB was only detected in those volunteers that produced LUNU.^21^ However, other metabolic steps and a possible explanation for the variability observed remained unexplored in that study.

Therefore, in the present study, we aimed to (i) decipher the metabolism of RSV by the human gut microbiota and (ii) identify the possible presence of metabotypes consistently associated with this metabolism in a group of healthy volunteers (*n* = 195), combining in vivo determinations with in vitro studies to confirm catabolic steps and specific enzymatic activities.

## Materials and Methods

### Reagents

HPLC-grade acetonitrile, dimethyl sulfoxide (DMSO), formic acid, and methanol were obtained from JT Baker (Deventer, The Netherlands). The following chemicals were purchased from Sigma-Aldrich (St. Louis, MO, USA): *trans*-resveratrol (3,5,4′-trihydroxy-*trans*-stilbene, resveratrol, RSV, ≥99%), *trans*-pinosylvin (3,5-dihydroxy-*trans*-stilbene, PINO, 98%), chrysin (97%), resorcinol (benzene-1,3-diol, 99%), 3-hydroxybenzoic acid (99%), 4-hydroxybenzoic acid (99%), 3′-hydroxyphenyl acetic acid (99%), 4′-hydroxyphenyl acetic acid (98%), 3-(3′-hydroxyphenyl)propanoic acid (98%), 3-(4′-hydroxyphenyl)propanoic acid (98%), β-glucuronidase (≥100,000 units/mL), and sulfatase (>10,000 units/g solid) from *Helix pomatia*. RSV 4′-*O*-sulfate, RSV 3-*O*-glucuronide, DHRSV 3-*O*-glucuronide, and RSV 3-*O*-sulfate were obtained as described elsewhere.^22^ 4-Hydroxy-*trans*-stilbene (4HST, 98%) was obtained from ThermoFisher Sci. (Madrid, Spain). Ultrapure Millipore water was used throughout the study.

### Chemical Synthesis and Spectroscopy Data of RSV-Derived Metabolites

^1^H NMR chemical shifts are reported relative to tetramethylsilane and referenced via residual proton resonances of the corresponding deuterated solvent. ^1^H NMR spectra were recorded at 25 °C on Avance 300 and 400 MHz instruments (Bruker, Karlsruhe, Germany). Coupling constants (*J*) are expressed in Hz. The abbreviations for the coupling patterns are the following: br, broad; s, singlet; d, doublet; t, triplet; q, quadruplet; and m, multiplet.^21^

DHRSV (>97%) was synthesized as previously described.^23^ Data for DHRSV: ^1^H NMR (400 MHz, DMSO-*d*_6_): δ 9.11 (s, 1H), 9.02 (s, 2H), 6.99 (d, *J* = 8.4 Hz, 2H), 6.65 (d, *J* = 8.5 Hz, 2H), 6.06 (d, *J* = 2.1 Hz, 2H), 6.02 (t, *J* = 2.1 Hz, 1H), 2.64 (m, 4H).

4-Hydroxydibenzyl (or 4-(2-phenylethyl)phenol, 4HDB; >97%) was synthesized according to Camaioni and Franz.^24^ Data for 4HDB: ^1^H NMR (400 MHz, DMSO-*d*_6_): δ 9.12 (s, 1H), 7.30–7.12 (m, 5H), 6.99 (d, *J* = 8.5 Hz, 2H), 6.64 (d, *J* = 8.4 Hz, 2H), 2.84–2.71 (m, 4H).

3-Hydroxydibenzyl (or 3-(2-phenylethyl)phenol, 3HDB; >97%) was synthesized as described elsewhere.^25^ Data for 3HDB: ^1^H NMR (300 MHz, CDCl_3_): δ 7.34–7.25 (m, 2H), 7.24–7.12 (m, 4H), 6.77 (dd, *J* = 7.6, 1.0 Hz, 1H), 6.71–6.65 (m, 2H), 4.71 (s, 1H), 2.96–2.83 (m, 4H).

3,4′-Dihydroxydibenzyl [or 3-(4-hydroxyphenethyl)phenol, LUNU; >97%] was synthesized following the procedure described by Ali et al.^26^ Data for LUNU: ^1^H NMR (300 MHz, CDCl_3_): δ 7.15 (td, *J* = 7.6, 0.7 Hz, 1H), 7.07–7.01 (m, 2H), 6.78–6.72 (m, 3H), 6.69–6.63 (m, 2H), 4.66 (s, 1H), 4.61 (s, 1H), 2.83 (br s, 4H).

Dihydropinosylvin (or 3-hydroxy-(5-phenethyl)phenol, DHP; >97%) was synthesized as previously reported.^26^ Data for DHP: ^1^H NMR (400 MHz, CDCl_3_): δ 7.32–7.26 (m, 2H), 7.20 (m, 3H), 6.25 (d, *J* = 2.2 Hz, 2H), 6.19 (t, *J* = 2.2 Hz, 1H), 4.75 (s, 2H), 2.92–2.85 (m, 2H), 2.84–2.78 (m, 2H).

Finally, 3,4′-dihydroxy-*trans*-stilbene (or 3-(4-hydroxystyryl)phenol, DHST, >97%) was synthesized as described elsewhere.^27^ Data for DHST: ^1^H NMR (400 MHz, DMSO-*d*_6_): δ 9.56 (s, 1H), 9.36 (s, 1H), 7.41 (d, *J* = 8.5 Hz, 2H), 7.13 (t, *J* = 7.8 Hz, 1H), 7.03 (d, *J* = 16.3 Hz, 1H), 6.98–6.88 (m, 3H), 6.76 (d, *J* = 8.5 Hz, 2H), 6.63 (d, *J* = 6.8 Hz, 1H) ppm.

### Volunteers and Study Design

This dietary intervention was approved (reference PI-042) by IMDEA-Food (Madrid, Spain) and the Spanish National Research Council’s Bioethics Committee (Madrid) within the MetaboGut Project (PID2019-103914RB-I00; MCIN, Spain). The trial followed the ethical guidelines outlined in the Helsinki Declaration of 1975 and its amendments. Healthy volunteers over 18 years old were recruited. Exclusion criteria involved the intake of resveratrol-containing foodstuffs, including red wine, peanuts, and pistachios (a list of foods and derivatives was provided to the volunteers). In addition, antibiotics (within a month before the study), pregnancy/lactation, history of smoking, diagnosed chronic illness, taking medication or food supplements (within a month before the study), previous gastrointestinal surgery, habitual consumption of more than 20 g of alcohol/day, being a vegetarian, or being on a weight loss regimen were included in the exclusion criteria. The study was fully explained to the volunteers, who provided written informed consent before participating. Three days before and during the intervention, the participants consumed a low-polyphenol diet, supervised by a nutritionist, and based on grilled meat-fish, rice, pasta, low-fat cheese, and bread, with a reduced contribution (1 portion/day) of the following: fruits and vegetables, legumes, nuts and seeds, juices, olive oil, coffee, and tea. The volunteers consumed one daily hard gelatin capsule containing 150 mg of RSV (98% purity) from *Polygonum cuspidatum* in the evening for 7 days. The capsules were manufactured by Laboratorios Admira S.L. (Alcantarilla, Murcia, Spain) following the requirements of the European Union’s good manufacturing practices. No placebo was included since this trial was not designed to test the effectiveness of a compound or evaluate changes in specific clinical variables. Since there was no clinical variable to accurately power (minimum sample size) for this type of study, the sample size was based on previous stratifying studies dealing with the gut microbiota-associated metabotyping of individuals.^2,13,17,28^

### Fecal Cultures

Twelve participants (6 males and 6 females as normoweight subjects) provided baseline stool samples. The volunteers were chosen after in vivo analyses to confirm catabolic steps in vitro. Fecal suspensions and culturing experiments were conducted as previously described.^29^ Samples were processed under anoxic conditions in an anaerobic chamber (Concept 400, Baker Ruskin Technologies Ltd., Bridgend, South Wales, UK) with an atmosphere of N_2_/H_2_/CO_2_ (85:5:10) at 37 °C. Briefly, aliquots were prepared with 10 g of stool samples and diluted with l-cysteine hydrochloride-supplemented nutrient broth using a stomacher for homogenization. Filtered suspensions were inoculated into Wilkins-Chalgren anaerobe medium (Oxoid, ThermoFisher Sci., Madrid, Spain) containing l-cysteine. The suspensions contained 30 μM of each standard dissolved in DMSO (0.6% DMSO in the final culture medium). The compounds (RSV, PINO, DHRSV, DHP, DHST, LUNU, 4HDB, and 4HST) were individually added to the broth and incubated under anoxic conditions in the anaerobic chamber described above. Fecal inocula from each volunteer plus broth with no compounds and each compound plus medium with no fecal inocula were used as controls. Three replicates were carried out using each fecal suspension and compound. The recovery of all metabolites assayed in vitro was >95%. Preliminary fecal incubations were carried out at different times, that is, 1, 2, 4, 7, and 10 days. Finally, all the incubations were performed for 7 days. After 7 days, samples were collected, processed, and further analyzed by UPLC-QTOF-MS and (or) gas chromatography–mass spectrometry(GC–MS) analyses.

### Sampling Procedure and Processing

The urine samples (baseline and after 7 days of RSV consumption) were centrifuged, filtered through a 0.22 μm PVDF filter, and diluted with water containing 0.1% formic acid. Diuresis was standardized by measuring the urinary excretion of creatinine, as previously reported.^30^ Besides, as described elsewhere, urine samples were treated overnight with glucuronidase and sulfatase to deconjugate phase-II conjugated metabolites.^30^ Non-hydrolyzed urine samples were analyzed by UPLC-QTOF-MS and hydrolyzed samples by UPLC-QTOF-MS and GC–MS. Stool samples were also provided before and after RSV consumption for 7 days and processed as previously described.^31^ Fecal samples from fermentation experiments were extracted with ethyl acetate plus formic acid and processed as reported elsewhere.^29^ Both fecal and in vitro-fermented fecal samples were analyzed by UPLC-MS-QTOF and GC–MS. In the case of GC–MS, urine, feces, and fecal fermentation samples were analyzed with and without derivatization. For derivatization, the evaporated residues of processed samples were dissolved in 30 μL of pyridine and converted to trimethylsilyl derivatives by adding 30 μL of *N*,*O*-bis-(trimethylsilyl) trifluoroacetamide containing 1% trimethylchlorosilane as previously described.^21^ In the non-derivatized samples, the residues after speed vacuum evaporation were dissolved in acetone and injected into the GC–MS equipment.

### UPLC-QTOF-MS Analyses

A previously validated method (linearity, precision, accuracy, limits of detection, and quantification) was used to analyze RSV and derived metabolites.^30^ Briefly, the analyses were performed on an Agilent 1290 Infinity UPLC system coupled to a 6550 accurate-mass quadrupole-time-of-flight (QTOF) mass spectrometer (Agilent Technologies, Waldbronn, Germany) using an electrospray interface (Jet Stream Technology), using chrysin as an internal control of the ionization signal. Spectra were acquired in the *m*/*z* range of 100 to 1100 in a negative polarity mode and at an acquisition rate of 1.5 spectra/s. Data were processed using Mass Hunter Qualitative Analysis software (version B.06.00, Agilent), which lists and rates possible molecular formulas consistent with the accurate mass measurement and the actual isotopic pattern. A target screening strategy was applied to qualitatively analyze possible metabolites that could be present after RSV consumption. In addition, targeted MS/MS analysis provided additional information to achieve a reliable compound identification. MS/MS product ion spectra were collected at *m*/*z* 50–800 range using a retention time window of 1 min, collision energy of 20 V, and an acquisition rate of 4 spectra/s.

### GC–MS Analyses

As recently described, samples were analyzed using an HP 8890 gas chromatograph with an HP 5977B mass selective detector (Agilent).^21^ An HP5-MS (30 m × 0.25 mm in inner diameter and a film thickness of 0.25 μm) phase capillary column was used with helium as a carrier gas at a constant rate of 1 mL/min, and the temperature of the injector and MS source was maintained at 200 °C. The samples were analyzed in a splitless mode. The MS was operated in the electron ionization mode with an ionization energy of 70 eV, and the mass spectrum was acquired in a positive electron impact (70 eV). Two methods with different MS parameters were performed depending on the metabolites. First, the metabolites 3HDB and 4HDB were analyzed without silylation. In this case, the selected ion monitoring acquisition type was chosen for the 198 and 107 *m/z* ions, segment retention times were fixed between 21 and 24 min in high-resolution mode, dwell time was set at 25 ms, and cycle time of 13.96 Hz, and finally, an electron multiplier voltage (EMV) of 1700 was calculated. The second methodology was used for silylated metabolites, that is, RSV, DHRSV, PINO, LUNU, DHP, DHST, and 4HST. In this case, the MS parameters were as follows: full scan mode acquisition type with a threshold of 1000, scan mass range from 50 to 800 Da at 2.0 scan/sec, a scan speed of 1.562 u/s, a cycle time of 502.60 ms, and finally, a calculated EMV of 1360.^21^

### Identification and Quantification of Metabolites

Direct comparison with available standards was used to identify different metabolites. In addition, their spectral properties, molecular masses, and fragmentation patterns were used to confirm the identification. These criteria were also used to identify some metabolites tentatively when no standards were available. For example, in the absence of standards, tentative identification was approached for LUNU and DHST conjugates, whose exact masses coincided with the expected metabolites, and further hydrolysis released the corresponding free forms, which were identified with the available standards. In the case of tentative phase-II 4HDB conjugates [glucuronides and (or) sulfates], its presence in urine was assumed since free 4HDB was identified using the corresponding standard in hydrolyzed urine after enzymatic treatment. Besides, tentative identification was also approached in possible isomers with identical mass to available standards, for example, 4HST and 3-hydroxy-*trans*-stilbene (3HST), which only differed in the −OH position. Finally, the quantification of RSV and derived metabolites was determined by interpolation of the calibration curves obtained with their corresponding standards in the urine and fecal matrices. In addition, using extracted ion chromatograms (EICs) for area calculation and quantification reduced the possibility of misinterpreting overlapping peaks.

### Statistical Analyses

Quantification of metabolites was expressed as mean ± SD. The statistical analyses were carried out using the SPSS software, v. 27.0.1.0 (SPSS Inc., Chicago, IL, USA). A multinomial logistic regression model was applied to evaluate the relationship between metabotypes and age, BMI, and sex. Using MetaboAnalyst 5.0 (https://www.metaboanalyst.ca), a heatmap was performed with normalized urine LUNU conjugate values (log-transformed) after consuming RSV for 7 days to group high and low LUNU-producers. Data plots were performed using SigmaPlot 14.5 (Systat Software, San Jose, CA, USA). Statistical significance was set at *P < 0.05.

## Results

### Volunteers’ Characteristics

Table 1 shows the main participants’ characteristics. The volunteers (*n* = 195) were recruited in two locations in Spain, namely, Madrid (*n* = 91) and Murcia (southeast of Spain, *n* = 104) to address any possible influence of geographical location on metabotype distribution. All the recruited participants were Europeans, and the mean age ranged from 18 to 81 years (41.5 ± 14.4), the BMI from 18.5 to 44.6, and there were more females (63.6%) than males (36.4%) (Table 1). No volunteers reported any side effects after consuming RSV for 7 days.

**Table 1:** Demographic Characteristics of the Subjects Included in This Study (*n* = 195)

	values^a^		
characteristics	all (*n* = 195)	Murcia (*n* = 104)	Madrid (*n* = 91)
age (years)	41.5 ± 14.4 (18–81)	43.0 ± 14.2 (18–81)	39.6 ± 14.5 (18–76)
weight (kg)	71.5 ± 14.6 (36.5–127.3)	69.0 ± 12.3 (48–117)	74.4 ± 16.4 (36.5–127.3)
BMI (kg/m^2^)	25.7 ± 5.0 (18.5–44.6)	24.8 ± 4.5 (18.5–44.6)	26.7 ± 5.3 (19.3–41.6)
normal weight	115 (59%)	67 (64.4%)	48 (52.7%)
overweight	36 (18.5%)	24 (23.1%)	12 (13.2%)
obese	44 (22.5%)	13 (12.5%)	31 (34.1%)
sex (female/male)	124/71	72/32	52/39

a Values are shown as mean ± SD (and range).

**Figure 1:**
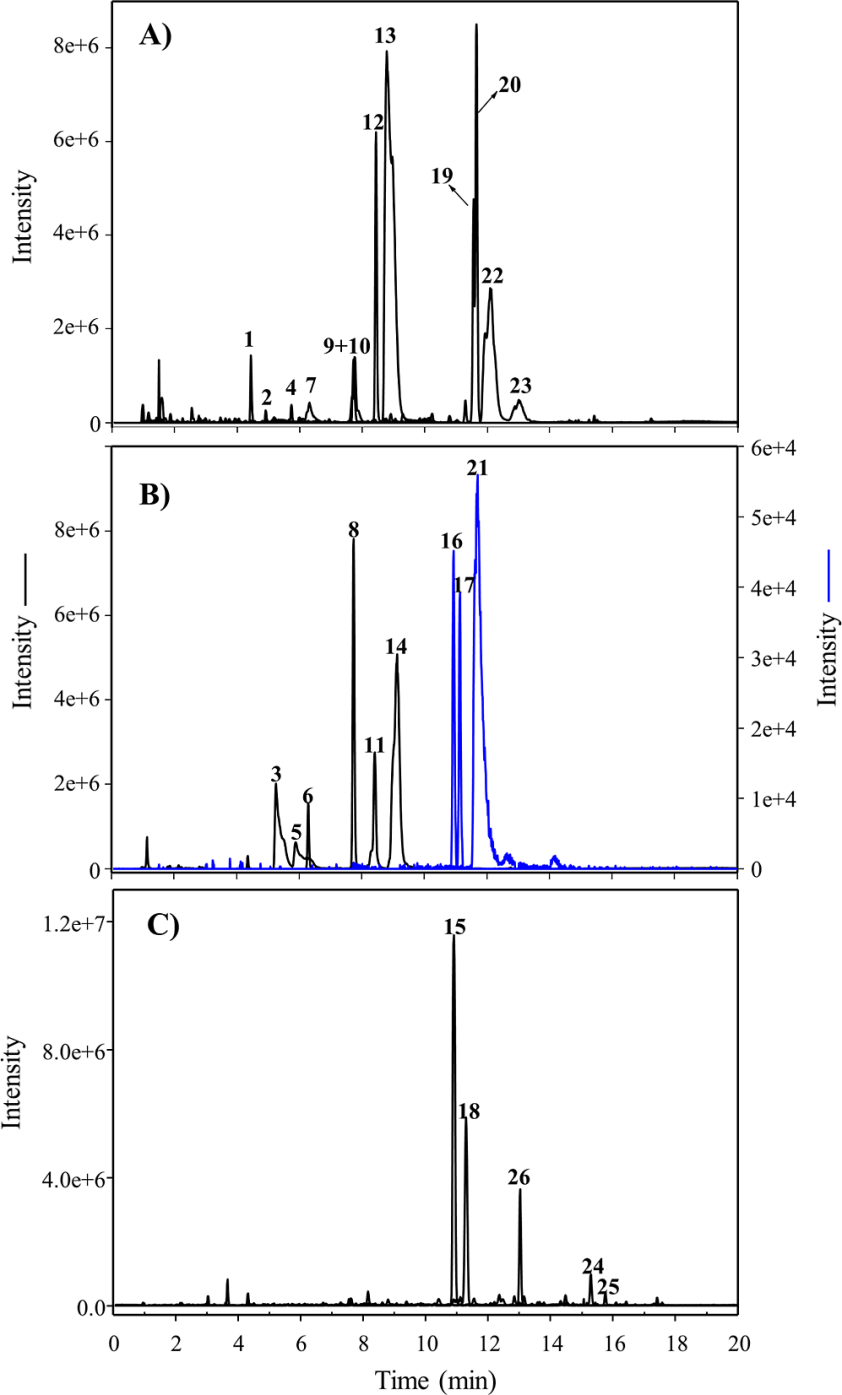
(A,B) UPLC-QTOF-MS EICs show the resveratrol (RSV)-derived metabolites in non-hydrolyzed urine from a LUNU-producer volunteer. (C) EICs show free RSV and its derived gut microbial metabolites in hydrolyzed urine from a LUNU-producer volunteer. Peak numbers are listed in Table 2.

The ionization of unconjugated metabolites from hydrolyzed urine and fecal samples was poor by UPLC-QTOF-MS, while the signal obtained by GC–MS proved to be better for detecting and quantifying these unconjugated metabolites. For this purpose, GC–MS analyses of silylated and non-silylated samples completed the metabolic profile in LUNU-producers (Table 3). Figures 3A and 3C show the GC–MS analyses of silylated and non-silylated standards, respectively. LUNU, *cis*-RSV, DHRSV, DHST, and RSV were detected in LUNU-producers (Figure 3B), confirming the same profile observed by UPLC-QTOF-MS (Figure 1C). Notably, the production of 4HDB, but not 3HDB, was observed in non-silylated samples of LUNU-producers (Figure 3D). As expected, only *cis*-RSV, DHRSV, and RSV were detected in LUNU non-producers (Figure 3E). The stilbenes PINO, 4HST, 3HST, and the dibenzyls DHP and 3HDB were not detected in any sample of LUNU-producers or non-producers (Figure 3 and Table 3).

**Figure 2:**
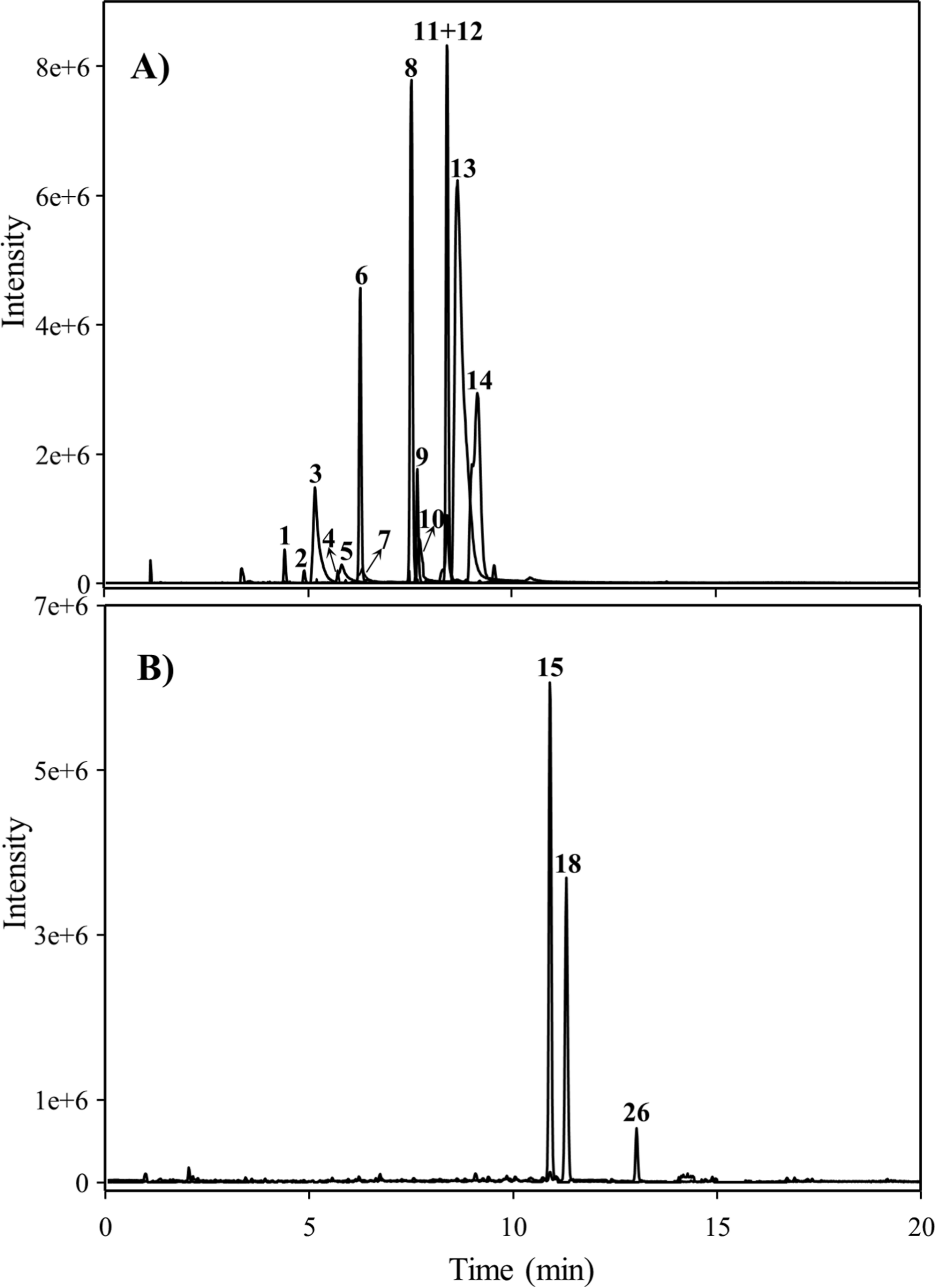
(A) EICs show resveratrol (RSV)-derived metabolites in non-hydrolyzed urine from a LUNU non-producer volunteer. (B) EICs show free RSV and its derived gut microbial metabolites in hydrolyzed urine from a LUNU non-producer. Peak numbers are listed in Table 2.

**Figure 3:**
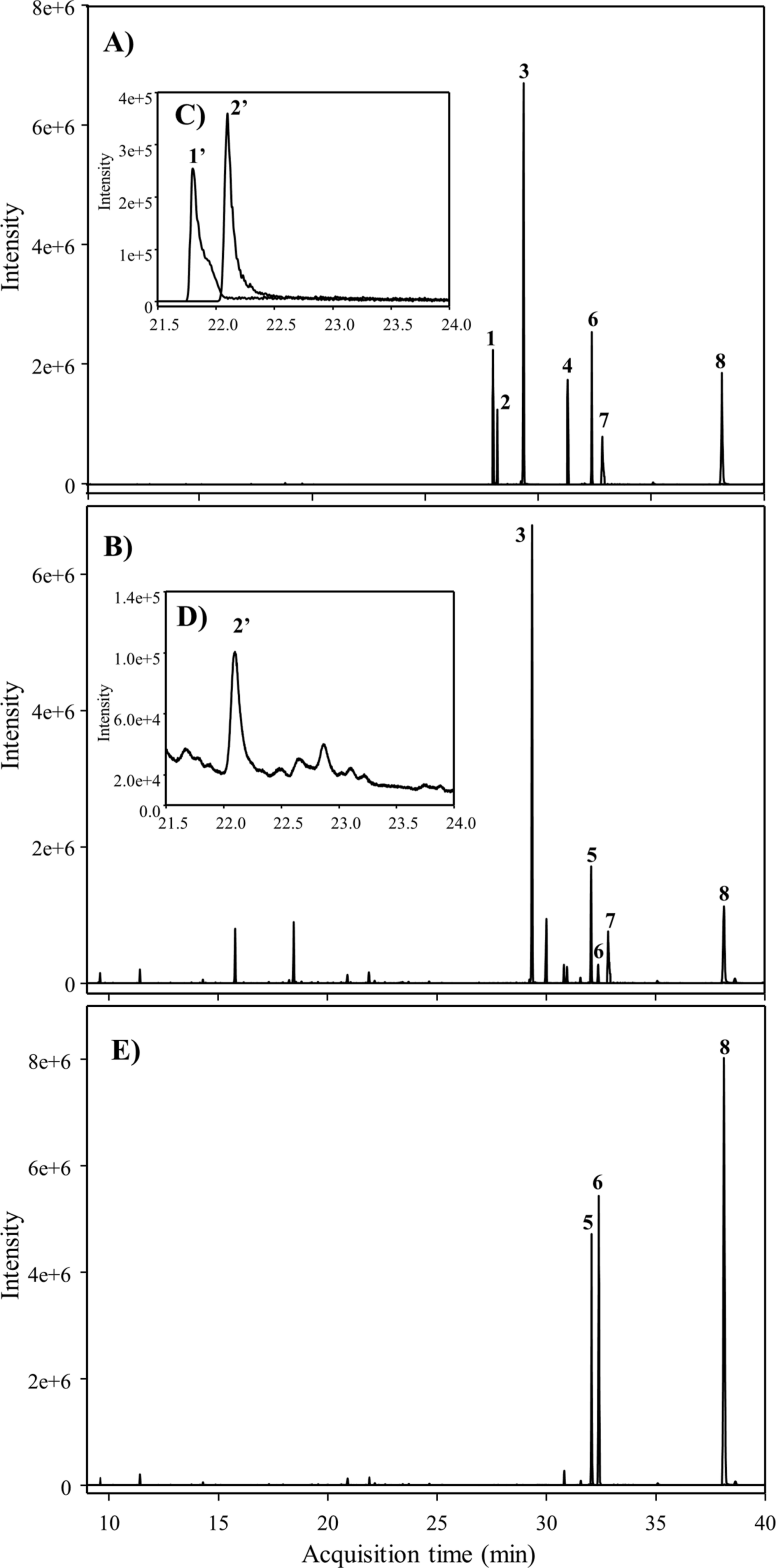
GC–MS EICs after silylation of (A) standards and (B) hydrolyzed urine from a LUNU-producer. Insets: GC–MS EICs of non-silylated (C) standards and (D) hydrolyzed urine from a LUNU-producer volunteer. (E) GC–MS EICs after silylation of hydrolyzed urine from a LUNU non-producer. Peak numbers are listed in Table 3.

**Table 2:** Resveratrol and Its Derived Metabolites in Urine, Feces, and (or) Fecal Cultures^a^

*N*°	compound	RT (min)	mass accuracy (*m*/*z*^*–*^)	molecular formula	error (ppm)	score	occurrence
UPLC-QTOF-MS (non-hydrolyzed samples)							
**1**	resveratrol (RSV) diglucuronide (isomer-1)^b^	4.43	579.1355	C_26_H_28_O_15_	1.18	98.87	U
**2**	RSV diglucuronide (isomer-2)^b^	4.90	579.1355	C_26_H_28_O_15_	0.06	99.66	U
**3**	RSV sulfoglucuronide (isomer-1)^b^	5.21	483.0603	C_20_H_20_O_12_S	–0.38	97.96	U
**4**	DHRSV diglucuronide^b^	5.73	581.1512	C_26_H_30_O_15_	0.56	99.18	U
**5**	RSV sulfoglucuronide (isomer-2)^b^	5.89	483.0603	C_20_H_20_O_12_S	0.15	97.69	U
**6**	RSV 4′-*O*-glucuronide^b^	6.28	403.1035	C_20_H_20_O_9_	–1.14	98.73	U
**7**	DHRSV sulfoglucuronide^b^	6.30	485.0759	C_20_H_22_O_12_S	0.6	98.64	U
**8**	RSV 3-*O*-glucuronide*	7.55	403.1035	C_20_H_20_O_9_	–1.99	97.07	U
**9**	DHRSV 4′-*O*-glucuronide^b^	7.69	405.1191	C_20_H_22_O_9_	1.21	98.39	U
**10**	RSV 4′-*O*-sulfate*	7.75	307.0282	C_14_H_12_O_6_S	–0.11	99.1	U
**11**	DHRSV 4′-*O*-sulfate^b^	8.42	309.0438	C_14_H_14_O_6_S	–0.96	99.01	U, F
**12**	DHRSV 3-*O*-glucuronide*	8.43	405.1191	C_20_H_22_O_9_	–0.02	97.09	U
**13**	RSV 3-*O*-sulfate*	8.77	307.0282	C_14_H_12_O_6_S	–1.95	96.76	U
**14**	DHRSV 3-*O*-sulfate^b^	9.05	309.0438	C_14_H_14_O_6_S	0.47	97.67	U, F
**15**	RSV*	10.79	227.0714	C_14_H_12_O_3_	–1.63	86.23	F
**16**	DHST-glucuronide (isomer-1)^b^	10.92	387.1085	C_20_H_20_O_8_	–1.82	97.51	U
**17**	DHST-glucuronide (isomer-2)^b^	11.12	387.1085	C_20_H_20_O_8_	–2.36	92.66	U
**18**	DHRSV*	11.35	229.0870	C_14_H_14_O_3_	–1.42	98.56	F, FC
**19**	LUNU glucuronide (isomer-1)^b^	11.55	389.1242	C_20_H_22_O_8_	0.24	99.38	U, F
**20**	LUNU glucuronide (isomer-2)^b^	11.65	389.1242	C_20_H_22_O_8_	–0.42	99.4	U, F
**21**	DHST sulfate^b^	11.69	291.0333	C_14_H_12_O_5_S	0.08	76^c^	U
**22**	LUNU sulfate (isomer-1)^b^	12.07	293.0489	C_14_H_14_O_5_S	–1.22	98.52	U, F, FC
**23**	LUNU sulfate (isomer-2)^b^	12.99	293.0489	C_14_H_14_O_5_S	–2.12	97.8	U, F, FC
**24**	LUNU*	15.34	213.0921	C_14_H_14_O_2_	–1.01	99.26	F, FC
**25**	DHST*	15.82	211.0765	C_14_H_12_O_2_	0.02	99.76	F, FC
UPLC-QTOF-MS (hydrolyzed urine)							
**15**	RSV*	10.94	227.0714	C_14_H_12_O_3_	–1.49	98.09	
**18**	DHRSV*	11.35	229.0870	C_14_H_14_O_3_	–1.58	98.63	
**26**	*cis*-RSV	13.02	227.0714	C_14_H_12_O_3_	1.38	97.96	
**24**	LUNU*	15.34	213.0921	C_14_H_14_O_2_	–1.35	98.86	
**25**	DHST*	15.83	211.0765	C_14_H_12_O_2_	–0.24	98.96	

a U, urine; F, feces; FC, fecal in vitro culture. *Identification using authentic standards.

b Conjugates tentatively identified (i) according to their exact molecular formula, high score (>90), low error (<5 ppm), and fragmentation pattern, and (ii) their disappearance after enzymatic hydrolysis with the simultaneous detection of the corresponding free metabolites (identified with standards).

c Although the score was below 90, the molecular mass was consistent with a sulfate derivative, and the metabolite disappeared after sulfatase hydrolysis. RSV, *trans*-resveratrol; DHRSV, dihydroresveratrol; DHST, 3,4′-dihydroxy-*trans*-stilbene; LUNU, lunularin.

**Table 3:** RSV and Related Metabolites Determined by GC–MS in Urine* and Feces After RSV Intake (*n* = 195) and (or) Fecal Cultures After Individual Incubation of RSV, DHRSV, LUNU, PINO, DHP, DHST, and 4HST (*n* = 12)^a^

*N*°	compound	RT (min)	mass	target ion	LOD; LOQ (nM)	occurrence	urine (μg/mg creatinine)^b^	feces (μg/g)^b^
silylated								
**1**	DHP	28.0	267	267	200; 500	ND		
**2**	4HST	28.1	268	268	200; 500	ND		
**3**	LUNU	29.3	358	179	50; 100	U, F, FC	88.2 ± 121.2	32.5 ± 28.7
**4**	PINO	31.3	356	356	100; 250	ND		
**5**	*cis*-RSV	32.0	444	444		U	274.0 ± 145.1^c^	
**6**	DHRSV	32.4	446	179	100; 250	U, F, FC	915.3 ± 654.8	34.1 ± 51.5
**7**	DHST	32.9	356	356	200; 500	U, F	11.2 ± 5.1	D
**8**	RSV	38.2	444	444	100; 250	U, F, FC	472.5 ± 269.0	1.4 ± 2.8
non-silylated								
**1′**	3HDB	21.8	198^d^	107	200; 500	ND		
**2′**	4HDB	22.1	198^d^	107	100; 250	U, FC	13.2 ± 30.5	

a U, urine; F, feces; FC, fecal culture; ND, not detected; D, detected but not quantified. *Hydrolyzed samples. 4HST, 4-hydroxy-*trans*-stilbene; LUNU, lunularin; DHRSV, dihydroresveratrol; DHST, 3,4′-dihydroxy-*trans*-stilbene; RSV (*trans*-resveratrol); 3HDB, 3-hydroxydibenzyl; 4HDB, 4-hydroxydibenzyl.

b Mean ± SD.

c Tentatively quantified as RSV.

d Mass without silylation.

### Unraveling the Catabolic Pathway of Resveratrol by the Human Gut Microbiota

**Figure 4:**
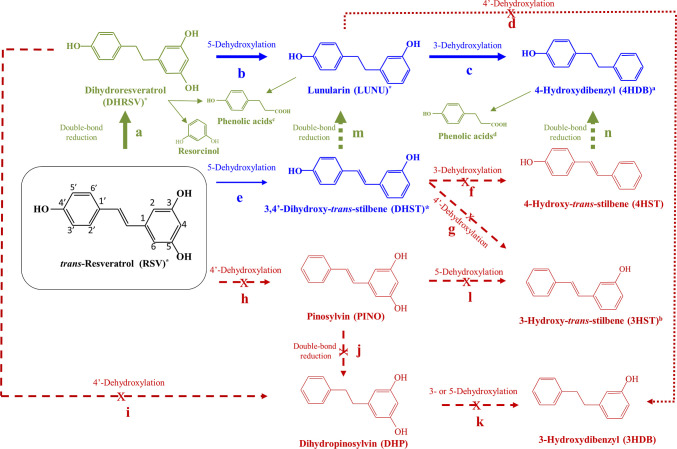
Proposed catabolism of resveratrol (RSV) by the human gut microbiota. Blue arrows and metabolites (LUNU, DHST, and 4HDB) are only present in LUNU-producers. Green arrows and metabolites (DHRSV, phenolic acid derivatives, resorcinol, and the double-bond reduction in **m** and **n** steps) are present in both metabotypes. Red arrows and metabolites (PINO, DHP, 3HST, 4HST, and 3HDB) were not present in vivo or in vitro. Thicker arrows designate more favored catabolic steps. Straight arrows refer to in vivo steps that were also confirmed in vitro. Dashed arrows refer to steps explored and confirmed in vitro. *Some phase-II conjugates were tentatively identified (Table 2). ^a^Phase-II 4HDB conjugates (glucuronides and sulfates) were assumed since the corresponding free 4HDB was detected in enzymatically hydrolyzed urine samples. ^b^3HST was discarded since the target ion 268 (similar to 4HST) was not detected in any sample (Table 3). ^c^Phenolic acids: 3′- and 4′-hydroxyphenyl acetic acids, 3-(3′-hydroxyphenyl)propanoic, and 3-(4′-hydroxyphenyl)propanoic acids, 3- and 4-hydroxybenzoic acids, and resorcinol. ^d^Phenolic acids: 4′-hydroxyphenyl acetic acid, 3-(4′-hydroxyphenyl)propanoic acid, and 4-hydroxybenzoic acid.

After analyzing the individuals’ metabolic profiles, we contacted the volunteers to provide stool samples for in vitro fermentation studies. Twelve volunteers (6 LUNU-producers and 6 LUNU non-producers) were selected. The in vitro experiments were not conceived to simulate the physiological conditions. Instead, the aims of the in vitro incubations were (i) to confirm the in vivo results and (ii) to explore if the metabolic machinery of the gut microbiota was capable of producing some metabolites that were not detected in vivo (Figure 4). Therefore, we assayed in vitro non-limiting incubation times (7 days) and concentrations of different precursors (30 μM) to favor the possible production of their corresponding metabolites.

First, the incubation of RSV in stool samples from LUNU-producers yielded DHRSV (Figure 4, step **a**), which was dehydroxylated at the 5-position to produce LUNU (step **b**). Then, a further 3-dehydroxylation yielded the metabolite 4HDB (step **c**). Notably, the metabolite 3HDB was not detected in any sample in vivo or in vitro, confirming the lack of dehydroxylase activity of the gut microbiota at the 4′-position of LUNU (step **d**). Furthermore, some DHST was also detected after RSV incubation (step **e**) but only in 2 of the 6 samples of LUNU-producers, showing similarities to those observed in vivo. Thus, this confirmed that the direct 5-dehydroxylation of RSV to produce DHST was a minor pathway, probably due to the presence of the double bond of the 4-styrylphenol core in RSV. In addition, no further dehydroxylation of DHST to yield 4HST was observed (step **f**), confirming the non-production of 4HST in LUNU-producers (Figure 3). Finally, since the target ion 268 from 4HST was not detected (Table 3), we could tentatively discard the isomer 3HST production (step **g**), which also reinforced the lack of dehydroxylation at the 4-position of DHST. Furthermore, the lack of 4-dehydroxylation of stilbenes and dibenzyls was also confirmed in vitro since neither PINO (step **h**) nor DHP (step **i**) were detected in any sample, confirming the in vivo results.

In LUNU non-producers, DHRSV was readily produced after RSV incubation (step **a**). However, LUNU and 4HDB were not detected in these samples (steps **b** and **c**), confirming that LUNU non-producers cannot dehydroxylate at the 3- or 5-positions, in agreement with in vivo results (Figures 2 and 3). Furthermore, as expected, 3HDB was not produced either (step **d**). In the same line, DHST, 4HST, or 3HST were not detected in LUNU non-producers. Finally, as in LUNU-producers, PINO and DHP were not detected, suggesting that the gut microbiota cannot dehydroxylate these dibenzyls and stilbenes at the 4- (or 4′)-position, independently of the capability to dehydroxylate at the 3- and 5- positions. Overall, all these results confirmed the in vivo findings.

The incubation of DHRSV and LUNU with samples from LUNU-producers confirmed steps **b**, **c**, and **d**, while DHRSV was not metabolized, and no other stilbene- or dibenzyl-related metabolites were detected in LUNU non-producers. Similarly, DHP was not produced in any sample from both metabotypes after incubation of DHRSV, confirming the in vivo results. In addition, 4HDB remained stable in the medium for 7 days, indicating no significant further degradation of this metabolite. Notably, the incubation of DHST yielded LUNU (step **m**) in both LUNU-producers and non-producers, confirming that the 4-styrylphenol reductase activity was common in both metabotypes. This fast reduction could explain the absence of 4HST in vivo and in vitro, despite the fact that LUNU-producers could theoretically dehydroxylate DHST at the 3-position to produce 4HST. Similarly, the incubation of 4HST only yielded 4HDB (step **n**), which was detected in both LUNU-producers and non-producers, further confirming the presence of the 4-styrylphenol reductase activity in both LUNU metabotypes.

Overall, the individuals from the metabotype LUNU-producers can dehydroxylate at the 5- and 3-positions (steps **a**, **b**, **c**, and **e**) but not at the 4-position (steps **d**, **g**, **h**, and **i**). This reaction is favored in dibenzyls (DHRSV and LUNU) versus stilbenes (DHST). On the other hand, individuals from the metabotype LUNU non-producers cannot dehydroxylate dibenzyls or stilbenes and only produce dibenzyls from stilbenes when the corresponding precursor is available, that is, RSV, DHST, or 4HST. However, this reduction requires the 4-styrylphenol core (not present in PINO).

As previously commented, the analysis of non-hydrolyzed urine samples yielded different dibenzyl and stilbene conjugates (Table 2), tentatively identified by their exact molecular formula, high score (>90), low error (<5 ppm), fragmentation pattern, and further glucuronidase/sulfatase treatment that released the corresponding free metabolites, which were identified with the available standards. Thus, phase-II conjugates from RSV and DHRSV were observed in both metabotypes, LUNU-producers and non-producers (Figures 1–3), while phase-II conjugates from LUNU and DHST (and presumably also from 4HDB) were only present in the LUNU-producer metabotype and confirmed indirectly after enzymatic hydrolysis of urine samples (Figures 1–3).

Finally, some phenolic acids and low-molecular-weight phenolic-derived metabolites were detected (results not shown). However, despite the low-polyphenol diet of individuals, these compounds were consistently present in all the samples, that is, urine and fecal samples before and after RSV consumption and fecal cultures of all compounds in controls and after fermentation with stools from both LUNU metabotypes. No clear differences were observed when comparing urine and feces before and after consuming RSV or between LUNU-producers and non-producers. In the case of fecal fermentations, there was a tendency to increase the amount of hydroxyphenyl benzoic, acetic, and propanoic acid derivatives as well as resorcinol, especially after incubation of DHRSV (results not shown). However, all these compounds were already present in the controls, preventing significant changes from being quantified in the current design.

### Distribution of LUNU Metabotypes

The distribution of LUNU metabotypes was 74.4% of LUNU-producers and 25.6% of LUNU non-producers when all the individuals were considered (*n* = 195). However, this distribution differed depending on the geographical location (*P* = 0.039), that is, 68.3% of LUNU-producers and 31.7% of LUNU non-producers in Murcia, while the distribution was 81.3% of LUNU-producers and 18.7% of LUNU non-producers in Madrid. We next explored the possible association of LUNU metabotype distribution with participants’ BMI, sex, and age, considering all the individuals and each specific location. No significant associations between metabotypes and BMI or age were observed in all individuals or by specific geographic location. However, there were more female LUNU non-producers than male non-producers in Murcia (40.3% females vs 12.5% males, *P* = 0.01) (Figure S1A, Supporting Information) compared to Madrid, where non-significant differences between sexes were observed (17.3% females vs 20.5% males, *P* = 0.69) (Figure S1B, Supporting Information). The significant differences were also observed when all the individuals were considered in the analysis (30.6% females vs 16.9% males, *P* = 0.037) (Figure S1C, Supporting Information), and the interaction between location and sex was also significant in the metabotype distribution (*P* = 0.027).

### High and Low LUNU-Producers

**Figure 5:**
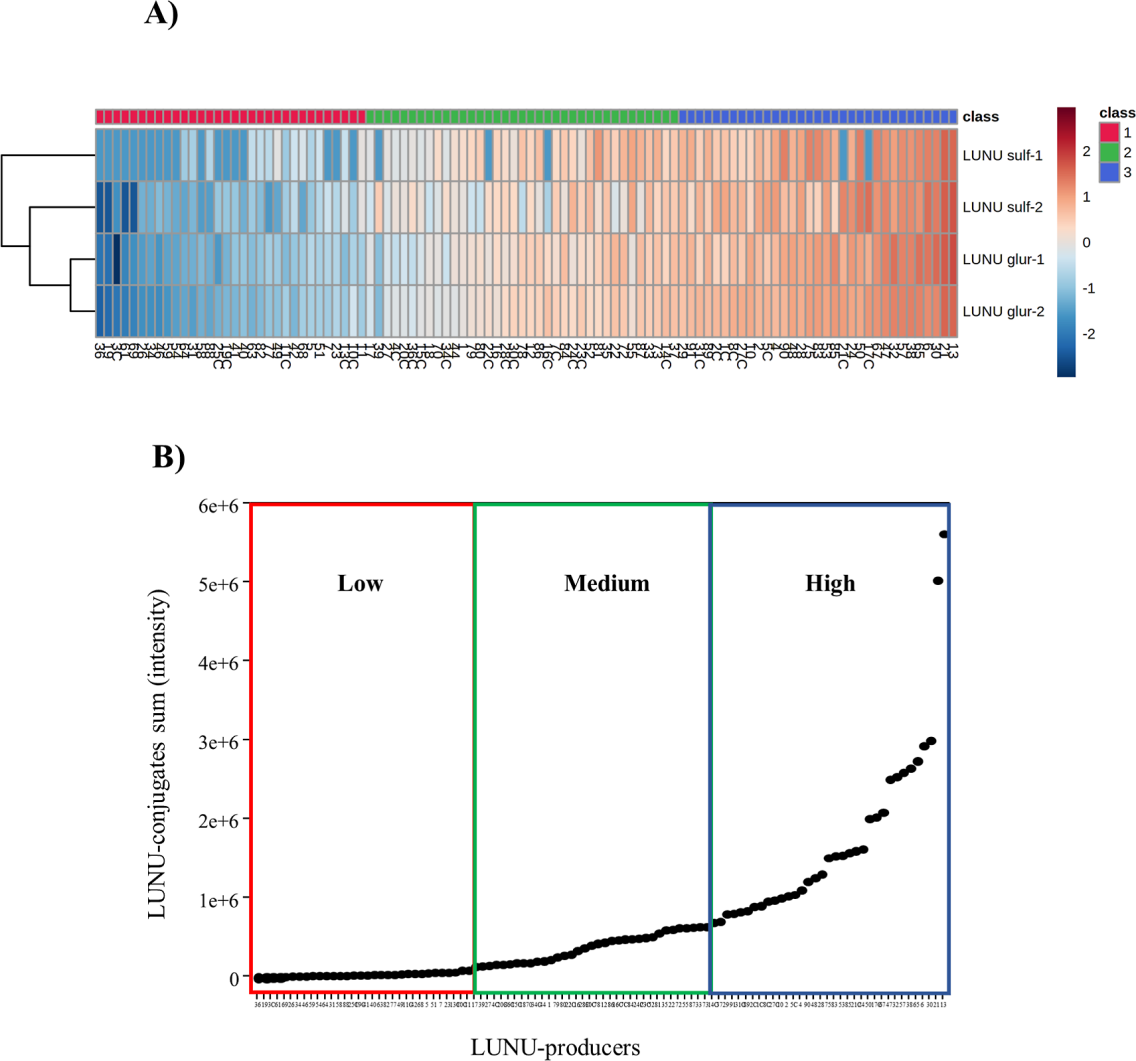
(A) Heatmap obtained after normalization of the values of LUNU metabolites by log transformation in the urine samples of LUNU-producers after consuming RSV for 7 days. Class: 1 (red), low producers, 2 (green), medium producers, and 3 (blue), high producers of LUNU conjugates. (B) Scatter plot showing the LUNU-producers’ clustering by high, medium, and low LUNU metabolite excretion in urine. LUNU conjugate values (EIC peak intensity) were standardized by urine creatinine concentration. The *X*-axis in both panels refers to the identification code of LUNU-producers.

The heatmap using urine log-transformed LUNU metabolite values (EIC peak intensities standardized by urine creatinine concentration) of LUNU-producers after consuming RSV for 7 days revealed the presence of high, medium, and low producers of the four LUNU metabolites, that is, glucuronides 1 and 2 and sulfates 1 and 2 (Figure 5A). In addition, these three groups were observed according to the production level of total LUNU metabolites (sum of glucuronides and sulfates), that is, low, medium, and high LUNU-producers (Figure 5B).

## Discussion

The catabolism of each specific (poly)phenol by the gut microbiota depends on the number, type, and position of specific functional groups, stereoisomerism, and polymerization degrees.^3,32,33^ These transformations depend on the individuals’ gut microbiota and critically affect (poly)phenol activity. There is notable interindividual variability in the metabolism and health effects of (poly)phenols. Thus, the stratification of individuals according to their metabotypes associated with (poly)phenol metabolism has been proposed to understand this variability, which could be crucial in the context of personalized nutrition.^2,4−7,17,18,34^ The urolithin and equol-ODMA metabotypes have been characterized in large cohorts and arise from particular bacterial species/strains with specific enzymatic machinery to catalyze characteristic reactions on the phenolic core.^2,14,35−38^

In the present study, we identify two metabotypes associated with RSV metabolism by the human gut microbiota for the first time, that is, LUNU-producers and LUNU non-producers. The gut microbial-derived metabolites of RSV metabolism in the LUNU-producer metabotype were DHRSV (and conjugates), DHST (and conjugates), and 4HDB (and presumably also its conjugates) (Figure 4, in blue). In contrast, DHRSV (and conjugates) were the only metabolites detected in the RSV metabolism by the LUNU non-producer metabotype (Figure 4, in green). In addition, phenolic acids and resorcinol were also detected in both metabotypes (Figure 4, in green). These metabolic profiles were consistently found in vivo and confirmed in vitro in both metabotypes. The crucial characteristic that allows identifying these metabotypes is the capability of LUNU-producers to dehydroxylate stilbenes and dibenzyls at the 3- and 5-positions, with much higher affinity for dibenzyls than stilbenes, while LUNU non-producers lack this dehydroxylase activity. The distinctive capacity to dehydroxylate (LUNU-producers) or not (LUNU non-producers) RSV and its derived dibenzyls is an intrinsic characteristic of the individuals, and it is not directly affected by external factors and thus, in the context of (poly)phenol metabolism, fits with the concept of metabotype associated with the gut microbiota.^2,7^

A gradient from high to low LUNU production was also identified, obviously only within the LUNU-producer metabotype. The existence of this production gradient of metabolites is a common feature in most (poly)phenols’ metabolism by the gut microbiota, reflecting the higher or lower abundance of bacteria capable of metabolizing the (poly)phenols and derived metabolites, and it is directly influenced by external factors (dietary pattern, motility of the gastrointestinal tract, food matrix, dose of (poly)phenol ingested, sample collection time, the sensitivity of the analytical procedure, etc.), which means that this gradient is not a genuine feature of each volunteer.^2,7,10^

We have confirmed here the metabolite 4HDB, but not 3HDB, produced after 3-dehydroxylation of LUNU (and thus, only present in LUNU-producers), which was recently identified in volunteers after consuming RSV.^21^ The metabolite 4HDB was consistently found in all LUNU-producers, that is, all showed 3- and 5-dehydroxylase activities. However, no complete conversion from LUNU to 4HDB was observed in any case. Therefore, taking into account the relative abundance of LUNU and the difficult determination of 4HDB,^21^ LUNU is a valid marker to stratify volunteers according to the ability of their microbiota to metabolize RSV. Finally, other significant findings of the present study are (i) the lack of dehydroxylase activity of the gut microbiota at the 4- (or 4′)-position in dibenzyls and stilbenes, and (ii) the specific 4-styrylphenol reductase activity present in both metabotypes. The double-bond reduction is probably the first and most universal reaction in gut microbiota’s catabolism of (poly)phenols, including hydroxycinnamic acids, flavonoids, stilbenes, and so forth.^3,20^ However, the existence of a specific 4-styrylphenol reductase activity for stilbenes, as evidenced by the inability to reduce pinosylvin lacking the 4-hydroxy group, is reported here for the first time to the best of our knowledge.

Some phenolic acids, including hydroxyphenyl benzoic, acetic, and propanoic acid derivatives, have been linked specifically to RSV metabolism by the gut microbiota in mice.^39^ However, in the present study, these metabolites were consistently found in all the samples, that is, in vivo and in vitro, including the controls. This agrees with previous studies that reported these metabolites as final degradation products in most (poly)phenols, including procyanidins, flavanones, hydroxycinnamic acid derivatives, anthocyanins, and so forth.^6,40^ Nevertheless, in the fecal fermentations, the increase of phenolic acids and resorcinol suggested the production of these metabolites in both LUNU metabotypes, especially after DHRSV incubation. Thus, these results suggest that scission or survival of the *meta*-dihydroxyphenyl ring and (or) the opening of aromatic rings might occur in DHRSV to yield these metabolites. Overall, our results suggest that producing these metabolites is not a hallmark of RSV metabolism. However, we acknowledge that the in vivo study’s design was not specifically addressed to characterize these metabolites, taking into account their promiscuous presence in all the samples. In addition, due to solubility problems, the low concentration of stilbenes and dibenzyls incubated in fecal fermentations could have prevented more significant differences versus controls in the production of these final metabolites.

LUNU metabotypes were not associated with BMI or age. However, the percentage of female versus male LUNU non-producers was higher in Murcia than in Madrid, even though there were more female than male participants in both locations. The explanation for this apparent association requires further research. In the case of the equol- or ODMA-producer metabotypes, no clear associations for any particular factor have been consistently detected, including dietary patterns, age, education level, anthropometric values, and race or ethnicity.^14^ In the urolithin metabotypes, aging is the main factor affecting metabotype distribution.^28^ In contrast, BMI, sex, dietary patterns, or other characteristics have not been conclusively associated with urolithin metabotypes.^2,28^

The dehydroxylase activity and substrate specificity of the class *Coriobacteriia* are known.^33^ For example, the specific dehydroxylase activity of some gut bacteria has been reported to be crucial in the metabolism of EA to yield urolithin metabotypes in which *Gordonibacter urolithinfaciens* can convert EA to urolithin C (3,8,9-trihydroxy-urolithin), a common metabolite in urolithin metabotypes.^41^ In contrast, *Ellagibacter isourolithinifaciens* can convert EA to isourolithin A, a characteristic metabolite of the urolithin metabotype B.^42^ Notably, the abundance of these species is very low in the urolithin non-producers (metabotype 0), supporting their inability to produce urolithins.^43^ In addition to EA, *G. urolithinfaciens* and *E. isourolithinifaciens* can metabolize caffeic, dihydrocaffeic, chlorogenic, and rosmarinic acids, but not other flavonoid and non-flavonoid (poly)phenols. Notably, these bacteria did not dehydroxylate or reduce the styrene double bond of RSV and other phenolics, suggesting a high substrate specificity.^44^ In contrast, the coriobacteria *Slackia equolifaciens*, *Adlercreutzia equolifaciens*, and *Adlercreutzia rubneri* strain ResAG-91T.8 can convert RSV into DHRSV,^9,45^ but the gut species capable of catalyzing the sequential dehydroxylations in stilbenes and dibenzyls remains to be elucidated.

As observed for other (poly)phenols, such as EA and isoflavones,^14,17^ it is plausible that human gut microbiotas with different compositions and functionalities might affect the outcome of trials also for RSV since the biological activity of the microbial-derived metabolites might differ from that of RSV.^46,47^ In this regard, recent studies have described low biological activities for DHRSV and LUNU in animal models, including caloric restriction mimetics,^46^ and their impact on insulin sensitivity.^47^ In contrast, other studies claim the high anticancer and anti-inflammatory activity of these metabolites.^48^ RSV, DHRSV, and LUNU conjugates were previously identified in plasma, urine, and mammary tissues of breast cancer patients.^29,49^ However, the lack of authentic standards of LUNU conjugates prevented their quantification in the bloodstream, urine, and breast tissues, which should be explored in further studies. Therefore, whether the sequential dehydroxylase activities of RSV that give rise to LUNU metabotypes significantly impact human health deserves further research. Similarly, our ongoing studies investigate the possible gut microbiota associated with LUNU metabotypes. Overall, we show here the most detailed metabolic pathway of RSV by the human gut microbiota reported to date and describe these novel metabotypes associated with RSV metabolism. This study could contribute to understanding the substantial human interindividual variability in the health effects of dietary (poly)phenols, including RSV.

## Supporting Information Available

Distribution of LUNU metabotypes according to the participants’ sex (PDF)

## Abbreviations

3HDB: 3-hydroxydibenzyl
3HST: 3-hydroxy-*trans*-stilbene
4HDB: 4-hydroxydibenzyl
4HST: 4-hydroxy-*trans*-stilbene
BMI: body mass index
CDCl_3_: deuterochloroform
DHP: dihydropinosylvin
DHRSV: dihydroresveratrol
DHST: 3,4′-dihydroxy-*trans*-stilbene
DMSO: dimethylsulfoxide
EA: ellagic acid
EIC: extracted ion chromatogram
EMV: electron multiplier voltage
eV: electronvolt
GC–MS: gas chromatography coupled to mass spectrometry
IUPAC: International Union of Pure and Applied Chemistry
*J*: coupling constants
LUNU: lunularin
MS: mass spectrometry
NMR: nuclear magnetic resonance
ODMA: *O*-desmethylangolensin
PINO: pinosylvin
PVDF: polyvinylidene fluoride
RSV: *trans*-resveratrol
SD: standard deviation
UPLC-ESI-QTOF-MS: ultra-high performance liquid chromatography coupled with electrospray ionization and quadrupole time-of-flight mass spectrometry
