# Supplementary material for: Lunularin Producers versus Non-producers: Novel Human Metabotypes Associated with the Metabolism of Resveratrol by the Gut Microbiota
Source: J Agric Food Chem. 2022 Aug 18;70(34):10521–31. doi: 10.1021/acs.jafc.2c04518 (PMC9449969; doi:10.1021/acs.jafc.2c04518)
Supplement: Supplementary file 1 — jf2c04518_si_001.pdf [file jf2c04518_si_001.pdf]

SUPPORTING INFORMATION

Lunularin Producers vs. Non-Producers: Novel Human Metabotypes Associated with the Metabolism of Resveratrol by the Gut Microbiota

Carlos E. Iglesias-Aguirre,[1] Fernando Vallejo,[1] David Beltrán,[1] Elena Aguilar-Aguilar,[2] Julio Puigcerver,[3] Mateo Alajarín,[3] José Berná,[3] María V. Selma,[1] and Juan Carlos Espín[1]*

[1]Laboratory of Food & Health, Research Group on Quality, Safety, and Bioactivity of Plant Foods, CEBAS-CSIC, 30100 Campus de Espinardo, Murcia, Spain; [2]Nutrition and Clinical Trials Unit, GENYAL Platform, IMDEA-Food Institute, CEI UAM + CSIC, Madrid, Spain; [3]Department of Organic Chemistry, Faculty of Chemistry, University of Murcia, 30100 Murcia, Spain.

***Corresponding author:** Juan Carlos Espín, Ph.D., Laboratory of Food & Health, Research Group on Quality, Safety, and Bioactivity of Plant Foods, CEBAS-CSIC, Campus de Espinardo Nº25, 30100 Murcia, Spain. E-mail: jcespin@cebas.csic.es

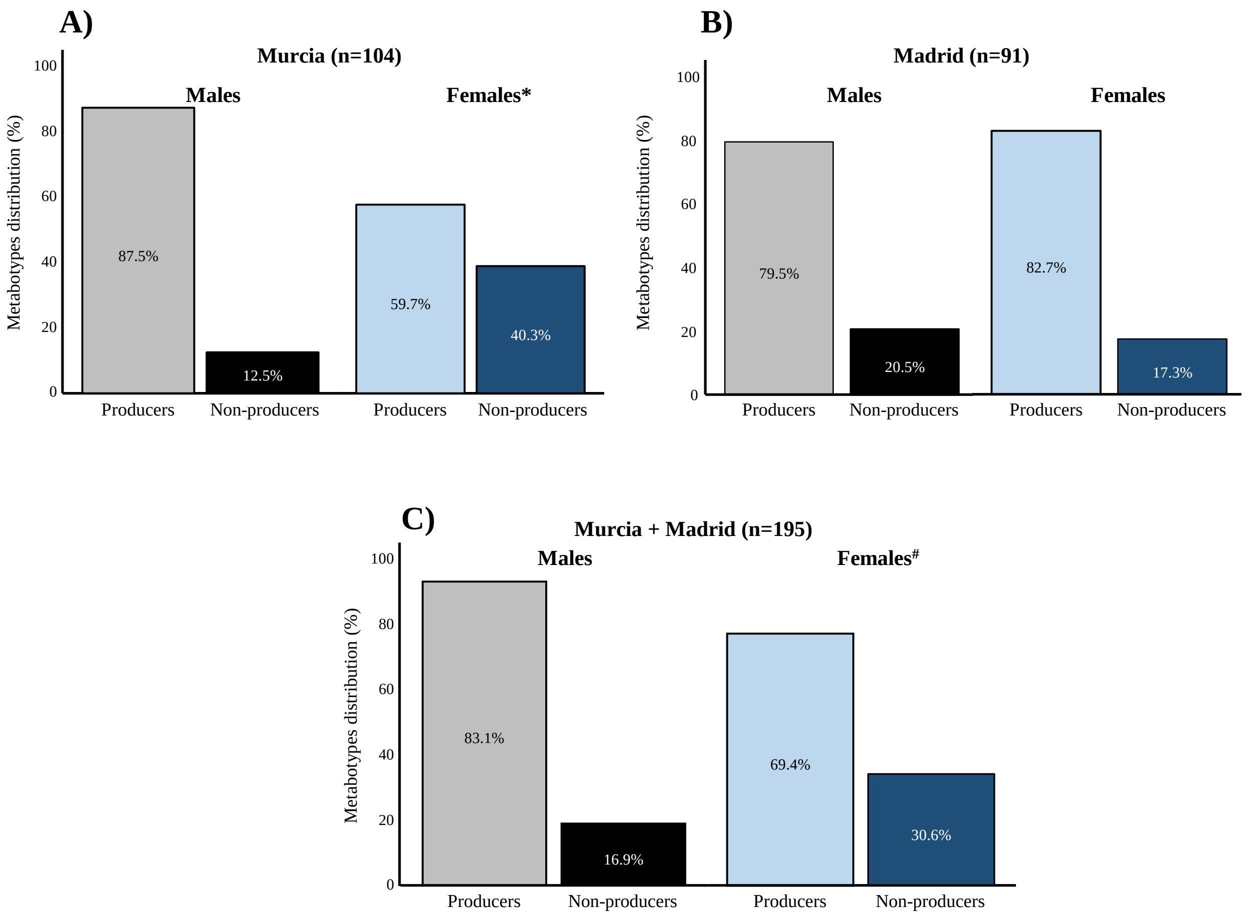

Figure 1S. Distribution of LUNU metabotypes according to the participants' sex. (**A**) Murcia, (**B**) Madrid, and (**C**) all the individuals. **P*=0.01 (females vs. males in Murcia) and #*P*=0.037 (females vs. males in Murcia+Madrid). Significant differences were determined using the Wald Chi-Square test (multinomial logistic regression model). The interaction between location and sex was also significant in the metabotypes distribution (*P*=0.027).
